# Upregulation of miR181a/miR212 Improves Myogenic Commitment in Murine Fusion-Negative Rhabdomyosarcoma

**DOI:** 10.3389/fphys.2021.701354

**Published:** 2021-08-06

**Authors:** Enrico Pozzo, Nefele Giarratana, Gabriele Sassi, Merve Elmastas, Theo Killian, Chao-chi Wang, Vittoria Marini, Flavio Ronzoni, Jason Yustein, Anne Uyttebroeck, Maurilio Sampaolesi

**Affiliations:** ^1^Stem Cell Institute Leuven, KU Leuven, Leuven, Belgium; ^2^Leuven Cancer Institute, University Hospitals Leuven, Leuven, Belgium; ^3^VIB KU Leuven Center for Cancer Biology, Leuven, Belgium; ^4^Human Anatomy Unit, Department of Public Health, Experimental and Forensic Medicine, University of Pavia, Pavia, Italy; ^5^Department of Biomedical Sciences, Humanitas University, Milan, Italy; ^6^Cancer and Hematology Center, Texas Children’s Hospital, Houston, TX, United States; ^7^Dan L Duncan Comprehensive Cancer Center, Baylor College of Medicine, Houston, TX, United States; ^8^Department of Molecular and Cellular Biology, Baylor College of Medicine, Houston, TX, United States; ^9^Integrative Molecular and Biomedical Sciences, Baylor College of Medicine, Houston, TX, United States; ^10^Department of Oncology, KU Leuven, Leuven, Belgium; ^11^Department of Paediatric Haemato-Oncology, University Hospitals Leuven, Leuven, Belgium

**Keywords:** rhabdomyosarcoma, skeletal muscle, murine model, pediatric cancer, microRNA, promyogenic signaling, promyogenic cocktail

## Abstract

Fusion-negative rhabdomyosarcoma (FN-RMS) is the most common soft tissue sarcoma of childhood arising from undifferentiated skeletal muscle cells from uncertain origin. Currently used therapies are poorly tumor-specific and fail to tackle the molecular machinery underlying the tumorigenicity and uncontrolled proliferation of FN-RMS. We and other groups recently found that microRNAs (miRNA) network contributes to myogenic epigenetic memory and can influence pluripotent stem cell commitments. Here, we used the previously identified promyogenic miRNAs and tailored it to the murine FN-RMS. Subsequently, we addressed the effects of miRNAs *in vivo* by performing syngeneic transplant of pre-treated FN-RMS cell line in C57Bl/6 mice. miRNA pre-treatment affects murine FN-RMS cell proliferation *in vivo* as showed by bioluminescence imaging analysis, resulting in better muscle performances as highlighted by treadmill exhaustion tests. In conclusion, in our study we identified a novel miRNA combination tackling the anti-myogenic features of FN-RMS by reducing proliferation and described novel antitumorigenic therapeutic targets that can be further explored for future pre-clinical applications.

## Introduction

Rhabdomyosarcoma (RMS) is the most common soft tissue sarcoma occurring in the pediatric population, accounting for ∼5% of all pediatric malignancies, and the overall event-free survival is less than 20% in patients with metastatic disease (Chen et al., 2019). RMS can be classified into two subtypes based on the histopathologic characteristics of the tumor, i.e., embryonal RMS (ERMS) and alveolar RMS (ARMS). Approximately 80% of ARMS present the chimeric proteins encoded from the fusion of PAX3 or PAX7 with FOXO1, while 20% are similar to ERMS in terms of clinical outcome (Skapek et al., 2019). ERMS, on the other hand, presents a number of causative mutations including *p53*, *Ras*, *Rb1*, and *Pten* (Rubin et al., 2011; Seki et al., 2015). It is now accepted that the fusion status better reflects the prognosis and tumor evolution with respect to tumor histology, thus fusion-positive (FP-RMS) and fusion-negative (FN-RMS) have replaced the current classification in order to identify the two RMS subtypes (Hibbitts et al., 2019; Rudzinski et al., 2021).

Given the expression of myogenic markers, it is hypothesized that RMS arises from myoblasts being restrained from fusion with syncytial muscle, thus leading to an invasive neoplastic phenotype (Kashi et al., 2015). Current treatment regimens include multimodality therapy involving surgery, high-dose chemotherapy and radiotherapy to resect the tumor, achieve remission and guarantee local control (Koscielniak et al., 1999). However, these therapeutic approaches fail to restore the myogenic propensity of FN-RMS and the persistence of chemoresistant cancer cells in the tumor are pushing research for the development of new therapies including the use of microRNAs (miRNAs). MiRNAs are small non-coding RNA molecules that bind to partially complementary mRNA sequences, resulting in target degradation or translation inhibition (Lim et al., 2005). Growing evidence is suggesting that miRNA loss-of-function can cause the progression of cancer, thus miRNA replacement therapies have emerged as promising treatment strategies for malignant neoplasms (Bonci et al., 2008; Esposito et al., 2014).

Recently, we have identified a pro-myogenic miRNA cocktail (PMC) by means of transcriptional and miRNA profile comparison between the mesodermal induced pluripotent stem cell-derived progenitors (MiPs) derived from human fibroblast and the skeletal muscle mesoangioblast (MAB)-derived MiPs (Giacomazzi et al., 2017). The identified miRNAs that were upregulated in MAB-derived MiPs were shown to rescue the myogenic intrinsic property of fibroblast-MiPs, thus paving the way for the potential contributions of the PMC in enhancing skeletal muscle differentiation in RMS, whereby the muscle differentiation is impaired.

In this study, we expand on the previously characterized murine model of FN-RMS by performing bulk RNA-seq on the cell lines derived from the model. Next, we hypothesize the use of PMC in murine FN-RMS aimed at restoring the myogenic propensity of the tumor. In order to do so, we first create a selection of PMC (s-PMC) and assess the s-PMC effectiveness on targeting proliferation reduction in murine RMS cells. Subsequently, we demonstrate its efficiency by means of syngeneic transplant of pre-treated murine FN-RMS in C57/Bl6 mice, showing cancer proliferation reduction upon treatment and functional amelioration during physical exercises.

Although deeper investigation is needed to tackle the quiescent tumor cells and metastatic niche and to rescue the tumorigenic phenotype, we identified a novel miRNA combination tackling the tumorigenic features of RMS by reducing proliferation and pinpointed novel antitumorigenic therapeutic targets that can be further explored for future pre-clinical applications.

## Materials and Methods

### Cell Culture

Human RD18 and murine KMR46 FN-RMS cell lines were cultured in high glucose Dulbecco’s Modified Eagles Medium (DMEM-HG) supplemented with 10% fetal bovine serum (FBS) and 1% Penicillin/Streptomycin (P/S). Cells were maintained under standard incubator conditions humidified atmosphere (95% air, 5% CO*2*, 37°C) and passaged twice weekly with 0.25% Trypsin-EDTA.

### Co-cultures

For co-culture experiments, cells were seeded at 1:20 KMR46/human MABs ratio on collagen-coated vessels in IMDM 15% FBS medium, 1% L-glutamine, 1% non-essential amino acids, 1% sodium pyruvate, 1% ITS, 1:10,000 basic FGF, 0.2% beta-mercaptoethanol. After 24 h, medium was changed and OptiMEM with miRNA-lipofectamine or lipofectamine was added to the wells. A total of 24 h after miRNA addition, cells were differentiated in DMEM 2% horse serum medium for 96–120 h in 5% O_2_/5% CO_2_ at 37°C.

### Reporter Cell Line

Cells were transfected with lentiviral particles expressing EF1a-eGFP-P2A-fLuc and selected with 1 μg/mL puromycin for 2 weeks. GFP was used as a standard gene expression tracer *in vitro*. Renilla luciferase (Rluc) was used as an optical reporter gene, upon coelenterazine administration, to detect the cell line engraftment *in vivo* via IVIS bioluminescence imaging (BLI).

### MiRNA Transfection

The miR-targeting cocktails were composed as follows: s-PMC, hsa-miR-181a-5p/-212-3p/-146b-5p/-132-5p; s-AMC, anti-hsa-miR-181a-5p/-212-3p/-146b-5p/-132-5p (all MISSION^®^ microRNA Mimics, Sigma-Aldrich). Before transfection, cells were seeded at 12–15.000 or 50.000 cell number in 24-wells or 6-wells, respectively. One day after plating, cells were transfected using 3 μl/mL Lipofectamine 2000 and 1.6 μl/mL of at 10 μM concentration, resuspended in OptiMEM-medium. After 60–65 h of transfection, cells were trypsinized, counted and either frozen or employed for subsequent experiments.

### Immunofluorescence

Following our adjusted protocol (Ronzoni et al., 2021), fixed cells were permeabilized with 1% BSA, 0.2% TritonX-100 in PBS for 5 min and then blocked in 10% donkey serum for 1 h. After 1.5 h of incubation with primary antibodies, diluted in 1% donkey serum, samples were washed three times with PBS, incubated with anti-mouse or anti-rabbit secondary antibody (1:1000) conjugated with 488 or 594 AlexaFluor fluorochromes (Invitrogen Milan, Italy) and nuclei were counterstained with Hoechst 33258 at 1 μg/ml (Sigma, Italy). Here follows the list of primary antibodies and relative dilutions: mouse anti-MyHC (DSHB #MF20), 1:3; rabbit anti-lamin A/C (Epitomics #2966-1), 1:600; mouse anti-Myogenin (Invitrogen #AB_10977211), 1:100; rabbit anti-MyoD (Thermo Fisher Scientific #MA5-12902), 1:100. Imaging was performed at Eclipse Ti microscope (Nikon) by means of Image-Pro Plus 6.0 software (Nikon).

### RNA Isolation and Quantitative Real-Time PCR

Total RNA of each sample was extracted using PureLink RNA Mini Kit (Ambion) and DNase I treatment was performed using the DNA-free kit (Ambion). Reverse transcription was performed using the Superscript III First-Strand Synthesis SuperMix (Invitrogen). A 384-well plate was prepared using Platinum SYBR Green qPCRSuperMix-UDG (Invitrogen) as SYBR Green on 1:5 diluted cDNA obtained from 1 μg total RNA (SybrGreen mix, SSIII cDNA production kit and RNA extraction kit from Thermo Fisher Scientific), using Viia7 384-plate reader (Thermo Fisher Scientific; final primer concentration, 100 nM; final volume, 10 μl; PGK, internal reference; thermal profile, 95°C 15 s, 60°C 60 s, 40×). Gene expression was analyzed in triplicate and normalized to the CT mean of *Rpl13a* and *Gapdh* as housekeeping genes. For the primer sequences, see Table 1.

**TABLE 1:** List of primer sequences used for gene expression analysis.

Gene name	Primer sequence
*Gdf15*	FW: GAGGTGCAGTCCCTGATTTG RV: AGTTCAGGCAAGTCACCCCT
*Myl6*	FW: CGTGGAGGTACCCTAACCCA RV: TTCAGCACCATCCGGACAAG
*Anxa3*	FW: ATGGCCTCTATCTGGGTTGGA RV: CAAGTCCTCTGATCGCTTTCC
*Tnnt1*	FW: CTGTGGTGCCTCCTTTGATT RV: GTCATCCTCTGCTCTCTTCTTTG
*Hand1*	FW: GCCTACTTGATGGACGTGCT RV: CAACTCCCTTTTCCGCTTGC
*Bmp6*	FW: CATTGCACCCAAAGGCTACG RV: TGGCATTCATGTGTGCGTTG
*Notch3*	FW: ACTCCTCCTCAGGGAGATGC RV: GTGGGGTGAAGCCATCAGG
*Zeb2*	FW: GCTGTGTTTGGTTGCTAGATCG RV: AGCGGATCAGATGGCAGTTC
*Myo1d*	FW: CCCAGTTGCTAATGAGCTGAA RV: AGTGACAAAAATTGCTCGGTCTT
*Acta1*	FW: CCCAAAGCTAACCGGGAGAAG RV: GACAGCACCGCCTGGATAG
*Acta2*	FW: GTCCCAGACATCAGGGAGTAA RV: TCGGATACTTCAGCGTCAGGA
*Myf5*	FW: TGCCATCCGCTACATTGAGAG RV: CCGGGGTAGCAGGCTGTGAGTTG
*Tnni2*	FW: GCACCTGAAGAGTGTGATGCT RV: TCTCCTTCTCAGATTCTCGGC
*Myog*	FW: GGGCCCCTGGAAGAAAAG RV: AGGAGGCGCTGTGGGAGTT
*Tnni1*	FW: GCTGAGAAGGTGCGTTACCTC RV: AGCTCTCGGCACAAGTCCT
*Myod*	FW: TACAGTGGCGACTCAGATGC RV: TAGTAGGCGGTGTCGTAGCC
*Myh1*	FW: CTCTTCCCGCTTTGGTAAGTT RV: CAGGAGCATTTCGATTAGATCCG
*Mrf4*	FW: ATTCTTGAGGGTGCGGATTTC RV: CCTTAGCAGTTATCACGAGGC
*Gapdh*	FW: AGGTCGGTGTGAACGGATTTG RV: TGTAGACCATGTAGTTGAGGTCA
*Rpl13a*	FW: CACCTGACCAAGTACCCTATTC RV: TTTGTGGTCTGCTGGGAAG

### RNA-Seq

RNA (>10 μg) samples were verified and processed by the Genomics Core (KU Leuven – UZ Leuven). RNA-sequencing libraries were constructed with the Lexogen library. Samples were indexed with unique adapters and pooled for single read (50 bp) sequencing in Illumina HiSeq2000. RNA-seq reads were aligned with TopHat v2.0.2 to the mouse genome mm10. Transcripts were assessed and quantities were determined by Cufflinks38. Differential expression levels were assessed using DESeq2. Gene Ontology Biological Process (GO:BP) pathways were identified using g:Profiler (Raudvere et al., 2019). Data has been deposited in GEO under accession code GSE175816.

### Transwell Migration Assay

To examine the migration potential following miR pretreatment, following our previous titration (Camps et al., 2020) 50,000 cells were seeded in the top chambers of a transwell in serum-free medium (#3422, Corning) as previously described (Balli et al., 2020). As chemoattractant, the lower compartment contained medium supplemented with 10% FBS. Cells were incubated for 24 h and cells that did not migrate through the pores were removed by a cotton swab. Filters were fixed in 4% formaldehyde solution and stained with 0.1% crystal violet. Five pictures per filter were taken and cell number was counted using ImageJ.

### Bioluminescence Imaging for *in vivo* Tumor Engraftment and Growth

Housing and all experimental animal procedures were approved by the Institutional Animal Care and Research Advisory Committee of KU Leuven (ECD #P089/2018). C57/Bl6 mice were injected in the femoral artery with either 1 × 10^5^ KMR46 Fluc+ cells (untreated group) or with 1 × 10^5^ KMR46 Fluc+ cells pretreated for 3 days with s-PMC (treated group). For the injection, cells were suspended in 50 μl saline water. Afterward, the mice were monitored through BLI every other day starting from day 7 after tumor injection for 10 days. For *in vivo* BLI scans, mice were placed in the flow chamber of IVIS^®^ Spectrum. Subsequently, 126 mg/kg of D-luciferin was injected subcutaneously (Breuls et al., 2021). Hence, consecutive frames were acquired until the maximum signal intensity was reached. Pulse/sec intensities were calculated by comparing the same ROI for all the animals, after subtracting the background signal coming from not injected mice.

### Treadmill Exhaustion Test

A group of five C57/Bl6 mice injected in the femoral artery with 1 × 10^5^ KMR46 Fluc+ cells (untreated group) and five C57/Bl6 mice injected with 1 × 10^5^ KMR46 Fluc+ cells pretreated for 3 days with s-PMC (treated group) underwent functional tests by the treadmill exhaustion test. The test was performed at day 7, 9, 11, and 13 after the beginning of the experiment (day 0). The electric shock frequency and intensity were pulses of 200 ms/pulse of electric current with 2 pulse/s repetition rate (3 Hz) and intensity (1.22 mA), as indicated by Giarratana et al. (2020). The mice were introduced to the treadmill belt and an adaptation time of 5 min was given before the recordings (motor speed set to zero, for 5 min). A training time of 2 min at 4 m/min was set. Later on, the motor speed was set to 7 m/min, with a 1 m/min increase and a constant uphill inclination of 20°, until exhaustion and >10 s stop. The mice were weighted right after every run. Speed (m/min), distance (m), and time (min and s) were registered and used for calculating the work of each run in J. The formula here applied was: Work (J) = body mass (kg) × gravity (9.81 m = s^2^) × vertical speed (m/s × angle) × time (s).

### Statistical Analysis

Sample size for *in vitro*/*in vivo* experiments was calculated by means of Sample Size Calculator (parameters: power, 0.80; alpha, 0.05).^1^ When applicable, sample size analysis was based on average values obtained from preliminary optimization/validation trials. Two-tailed unpaired Student’s *T*-tests analysis was performed. All statistical analyses were conducted using Prism v9.1.0 (GraphPad).

## Results

### RNA-Seq of Murine RMS Model Unveils Key Signature Compared to Satellite Cells

A novel murine model of FN-RMS has been recently described which showed high molecular homology between the genetically engineered murine model (GEMM) and human RMS (Nakahata et al., 2021). From this GEMM, the cell line KMR46 was derived (from here referred to as murine FN-RMS) which was demonstrated to preserve histological resemblance and metastatic potential with respect to its primary GEMM tumors (Nakahata et al., 2021).

**FIGURE 1:**
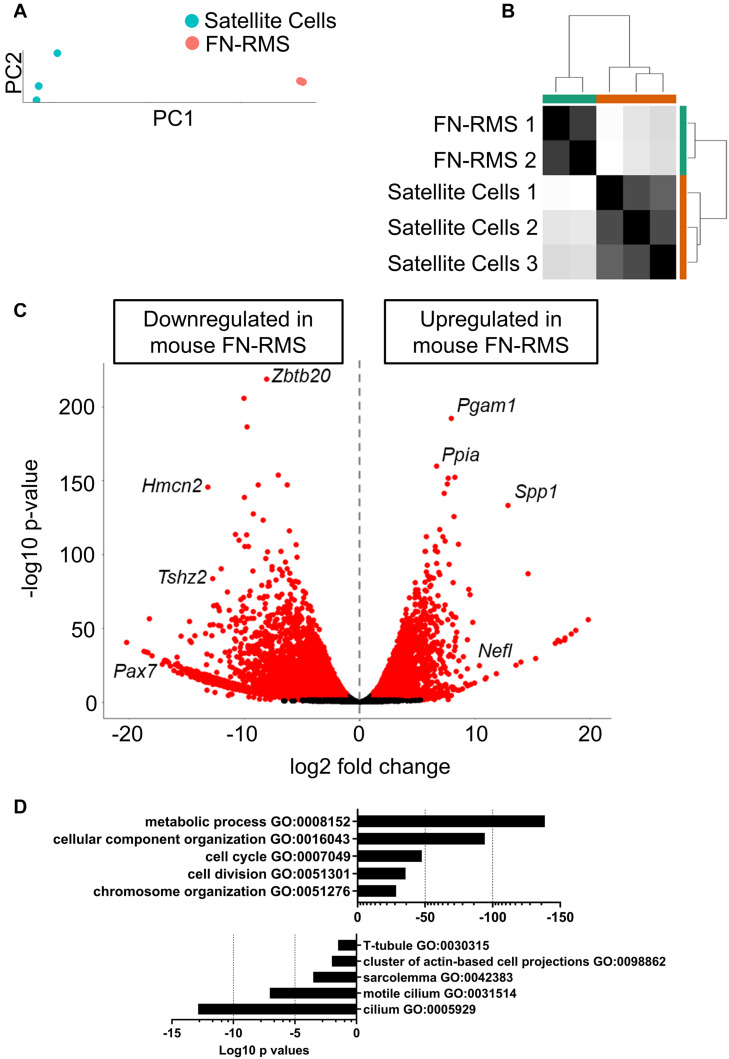
Identification of differentially expressed genes in murine FN-RMS cell lines. **(A)** Principal component of murine satellite cells (*n* = 3) and FN-RMS cell lines (*n* = 2), with PC1 accounting for 98% of the variance observed in the analysis. **(B)** Sample distance matrix showing closed proximity of the replicates. **(C)** Volcano plot of the up- and downregulated genes in murine FN-RMS compared to satellite cells. **(D)** Selection of Gene Ontology Biological Process pathways up- and down-regulated in murine FN-RMS compared to satellite cells.

In order to understand the key genes that distinguish the tumor from the normally developing skeletal muscle, we performed RNA-seq on the murine FN-RMS cell line and analyzed the differentially expressed genes with respect to the murine satellite cell dataset from Machado et al. (2017) using DESeq2 (Figure 1). We employed the T0 quiescent satellite cells for this comparison as it is largely accepted that a common progenitor of satellite cells could be the cell that transforms and becomes FN-RMS (Tiffin et al., 2003). PC (Figure 1A) and distance matrix (Figure 1B) analyses RNA-seq of all samples revealed similar clustering according to the cell of origin. We identified 15,276 downregulated genes and 5638 upregulated genes (Figure 1C). The discrepancy with respect to the number of genes shown to be downregulated compared to the upregulated ones can be sought in the hypermethylation status that characterizes FN-RMS, as previously described (Mahoney et al., 2012), which leads to the chromatin closure and subsequent reduced expression of several genes. Among the genes we identified, *Zbtb20*, *Hmcn2*, *Tshz2*, and *Pax7* were shown to be downregulated in murine FN-RMS compared to satellite cells. *Zbtb20* was previously described by Alonso-Martin et al. (2016) to be one of the key genes induced in muscle stem cells and involved in myogenic progression. The downregulation of *Pax7* and *Hmcn2*, on the other hand, points at both the deranged myogenic capacity as well as the poor cross-interactions with other cells, as *Hmcn2* is a key gene coding for ECM proteins which allows for efficient Pax7+ cell homing in skeletal muscle (Faralli et al., 2011; Wurmser et al., 2020). In the upregulated gene pool, *Pgam1* promotes cancer cell migration (Zhang et al., 2017), while *Ppia* has been shown to be correlated with poor prognosis in patients with hepatocellular carcinoma (Wang and Yu, 2019). *Spp1* (also known as osteopontin) has a key role in the physiology of skeletal muscle growth, and thus its upregulation may hint at its key role in the pathogenesis of FN-RMS (Pagel et al., 2014). Finally, *Nefl* has a key role in intracellular transport to axons and dendrites, and has been shown to be upregulated in several cancers including head and neck squamous cell carcinoma (Huang et al., 2014).

We then performed pathway analysis using g:Profiler (Figure 1D). Here, we identified pathways regarding metabolic process, cell cycle and chromosome organization as being upregulated in murine FN-RMS compared to satellite cells. Conversely, components of myotube formation such as T-tubule and sarcolemma were pathways found to be downregulated in murine FN-RMS. Intriguingly, cilium was one of the most downregulated pathways in murine FN-RMS, a structure that has been previously described to be present in quiescent satellite cells (Jaafar Marican et al., 2016) and its deregulation in myoblasts was shown to induce the formation of RMS (Fu et al., 2014).

### s-PMC Promotes Myogenic Commitment of Murine RMS *in vitro*

**FIGURE 2:**
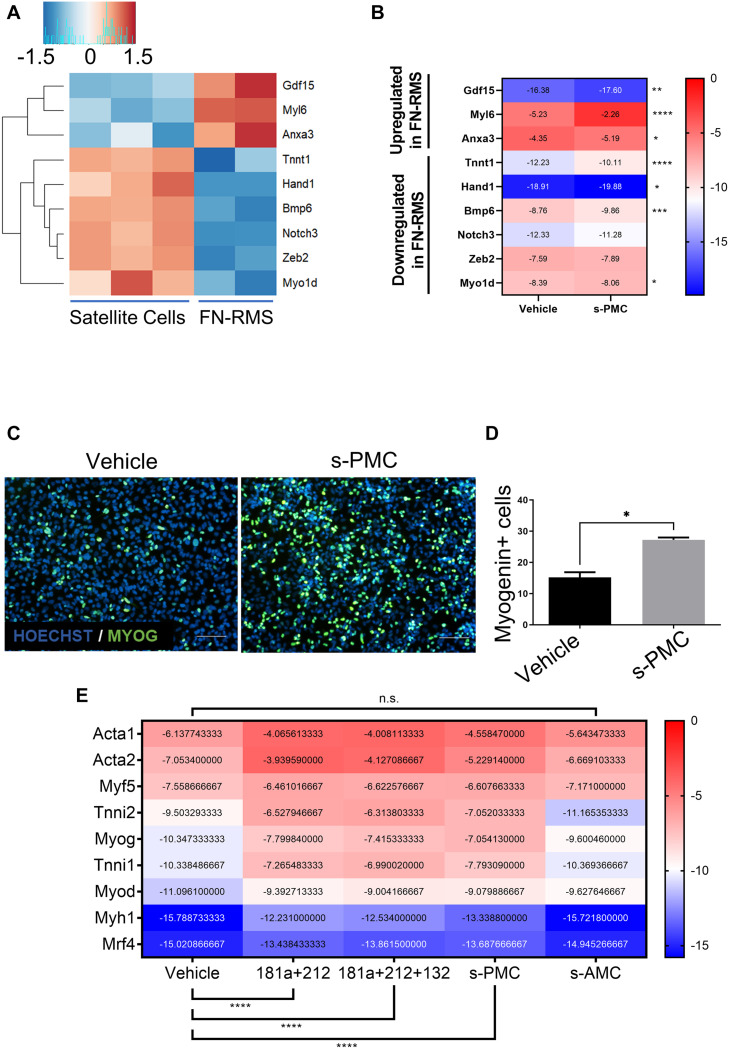
Selection of previously identified PMC improves myogenic potential in murine FN-RMS. Heatmaps of a selection of genes previously shown to be differentially expressed in MiPs upon PMC treatment **(A)** analyzed by RNA-seq and **(B)** confirmed in murine FN-RMS by qRT-PCR using s-PMC. **(C)** Myogenin expression in green and **(D)** quantification upon s-PMC treatment in FN-RMS cell lines compared to vehicle. Nuclei are counterstained in blue with HOECHST. s-PMC, Selected Promyogenic Cocktail. Scale bar, 100 μm. *p < 0.05. **(E)** Reductionist approach to identify the minimal combination of miRNAs from the s-PMC required to have the promyogenic effect. s-AMC, Selected Antimyogenic Cocktail. **p < 0.01; ***p < 0.001; ****p < 0.0001.

We sought to explore the possible use of previously identified PMC as a novel miRNA cocktail to promote myogenic commitment in murine RMS. We first performed *in silico* analysis by cross-checking the genes targeted by PMC in MiPs with the differentially expressed genes in murine FN-RMS (Figure 2A). We found an upregulation of *Anxa3, Gdf15*, and *Myl6* and downregulation of *Tnnt1, Myh1, Hand1, Notch3, Zeb2, Bmp6*, and *Myo1d* in murine FN-RMS compared to satellite cells. This indeed hints at the hypothesis of the use of PMC to promote myogenic commitment of murine FN-RMS and its subsequent loss of tumorigenic phenotype.

For our experiments, we decided to revisit the previously published PMC as it was specifically tailored for human MiPs. We therefore removed the antagomiR use, which reflected the miRNAs found to be upregulated in f-MiPs. Then, we checked for the murine sequences of the human miRNA mimics used in the PMC and found 4 out of 5 miRNAs (mmu-miR-181a-5p, mmu-miR-212-3p, mmu-miR-146b-5p, and mmu-miR-132-5p). We thus excluded miR-424 since the murine counterpart does not exist, and we named this newly identified PMC as selected PMC (s-PMC) (Supplementary Table 1). We then cross-checked the predicted target transcripts of the miRNAs with the upregulated genes in murine FN-RMS. Intriguingly, we found that 318 targets of miR-181a, 156 targets of miR-212, 138 targets of miR-146b and 12 targets of miR-132 are upregulated in murine FN-RMS compared to normal satellite cells (Table 2 and Supplementary Table 2). In order to address s-PMC effects on murine FN-RMS myogenic committment, we checked the previously identified miRNA targets and their expression after perturbation (Figure 2B). We then assessed myogenin localization, one of the key transcription factors involved in myogenic differentiation, after s-PMC treatment of murine FN-RMS (Figures 2C,D and Supplementary Figure 1). An increased percentage of myogenin was expressed in s-PMC-treated compared to vehicle-treated cells, which corroborated our hypothesis on the increased myogenic commitment of murine FN-RMS after s-PMC use. In order to identify the key miRNAs from the s-PMC responsible for the increased myogenic commitment, we performed in silico analysis using DIANA software to assess the different pathways targeted by the s-PMC (Vlachos et al., 2015; Supplementary Figure 2A). We then used a reductionist approach to identify the minimal selection of s-PMC components required to have a pro-myogenic effect (Figure 2E). To do so, we checked for the molecular expression of key myogenic genes (*Acta1, Acta2, Myf5, Tnni2, Myog, Tnni1, Myod, Myh1*, and *Mrf4*) at baseline levels, after s-PMC and after anti-myogenic cocktail. We then removed one and two miRNAs from the s-PMC and checked for the myogenic genes’ expression. Thus, we identified miR-181a/212 as the key combination required to have the pro-myogenic effect observed with the s-PMC, which was further confirmed by the *in silico* analysis (Supplementary Figure 2B).

**TABLE 2:** List of miRNAs in the s-PMC and the key targeted genes upregulated in FN-RMS.

List of miRNAs	Targeted genes
mmu-miR-181a-5p	Spry4, Ago2, Trim71, Mapk1, Lox, Klf6, Bcl2l11, Rassf1, Carm1, Slc2a3, Oxa11, Ipo8, Pdap1, Ttl, Aldh3a2, Oxa1, Tulp4, Wsb1, Gatm, Mpi, Rad21, Mtpn, Ano1, Arf6, Naa15, Nab1, Tmed4, Tgfbi, Ccnk, Fhl2, Igf2bp2, Kif3b, Ssr1, Snn, Itga3, Cbx4, Tfam, Caprin1, Rras2, Map1a, Pdgfra, Gpbp1, Dynll2, Plau, Actr2, Serbp1, Eif5a2, Ccnb1, Cdc7, Rab6b, Ccn1, Col16a1, Fgf7, Ccna2, Pkn2, Cdk7, Vcan, Idh1, Rab11a, Dlk2, Ifi44, Pak4
mmu-miR-212-3p	Mex3c, Rras2, Hmga2, Mapk1, Eif1b, Twist1, Nras, Lmnb2, Brca1, Melk, Uba3, Vcan, Caprin1, Sox11, Vdac2, Mob4, Igf2bp1, Trib2, Hdac3, Six4, Tmed7, Slit2, Mthfd2, Kif2a, Nab1, Rab11a
mmu-miR-146b-5p	Traf6, Kras, Rbm18, Eif4g2, Slc10a3, Abl2, Gid8, Vcan, Siah2, Tmx4, Parp1, Mlf2, Cdk5, Prx, Mcfd2, Ssr1, Axl, Vasn, Zyx, Tmx3, Fstl3, Pcdh1, Dstn, Dynll1, Ola1, Itga4
mmu-miR-132-5p	Irx2, Bivm, Tbc1d31, Klc2, Gbp3, Piga, Snx7, Slc20a2, Midn, Got2, Sema4b, Itga3

### miR-181a/212 Improve Differentiation While Targeting Proliferation and Migration

**FIGURE 3:**
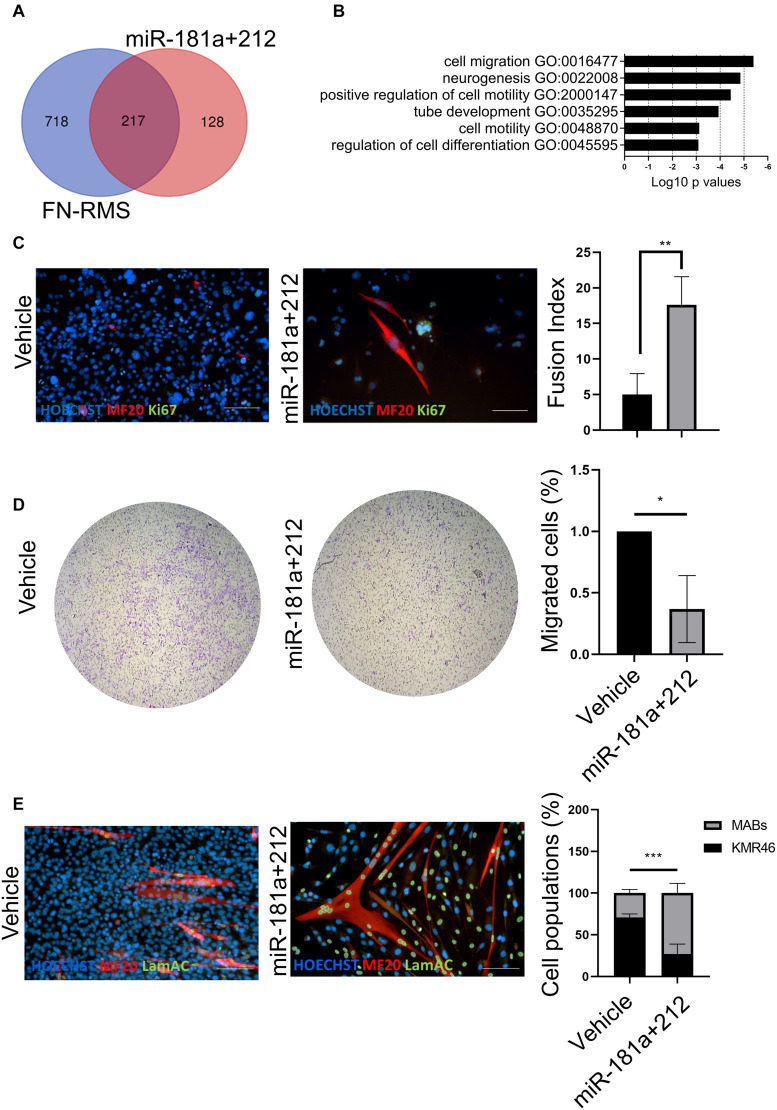
miR-181a/212 target differentiation, proliferation, and migration in murine FN-RMS. **(A)** Overlap and **(B)** selection of Gene Ontology Biological Process pathways upregulated in murine FN-RMS and targeted by miR-181a/212. **(C)** miR-181a/212 increases the myogenic commitment of murine FN-RMS as seen by the quantification of fusion index of MyHC+ myotubes. Nuclei are counterstained in blue with HOECHST. Scale bar, 100 μm. **(D)** Reduced migration capacity of murine FN-RMS upon miR-181a/212 treatment compared to vehicle. *p < 0.05. **(E)** Qualitative and quantitative effect of miR-181a/212 on murine FN-RMS proliferation in co-culture setting with human MABs compared to vehicle. Human nuclei were identified with anti-lamin A/C Abs in green. Nuclei are counterstained with HOECHST in blue. Scale bar, 100 μm. **p < 0.01; ***p < 0.001.

In order to identify the effects of miR-181a/212 on murine FN-RMS, we first performed *in silico* analysis to identify the pathway upregulated in RMS targeted by the miRNA combination (Figure 3A). We first checked the predicted gene targets of miR-181a/212 using miRDB (Chen and Wang, 2020) and identified the pathways upregulated in murine FN-RMS and targeted by miR-181a/212 using g:Profiler. We found migration, neurogenesis, and regulation of cell differentiation to be targeted by the miRNAs (Figure 3B). We therefore aimed at characterizing these effects *in vitro*. We first checked the regulation of cell differentiation by adding miR-181a/212 and differentiating the murine FN-RMS. We observed an increase of fusion index of MyHC+ myotubes in miRNA-treated compared to vehicle-treated FN-RMS cells (Figure 3C). We then assessed the migration potential of murine FN-RMS after miRNA treatment by means of transwell assay and indeed we observed a reduced migration potential after miRNA treatment (Figure 3D). Finally, as we wanted to assess the specificity of this cocktail in targeting the proliferation of murine FN-RMS, we co-cultured tumor cells with human MABs and quantified the percentage of cell populations after miR-181a/212 using human specific lamin A/C antibody (Figure 3E). Indeed, we observed that healthy muscle cells were not negatively affected by the miRNA in terms of cell number and cell differentiation.

### miR-181a/212 Pre-treatment in FN-RMS Cells Reduces Tumor Size and Improve Running Performance in Tumor Bearing-Mice

**FIGURE 4:**
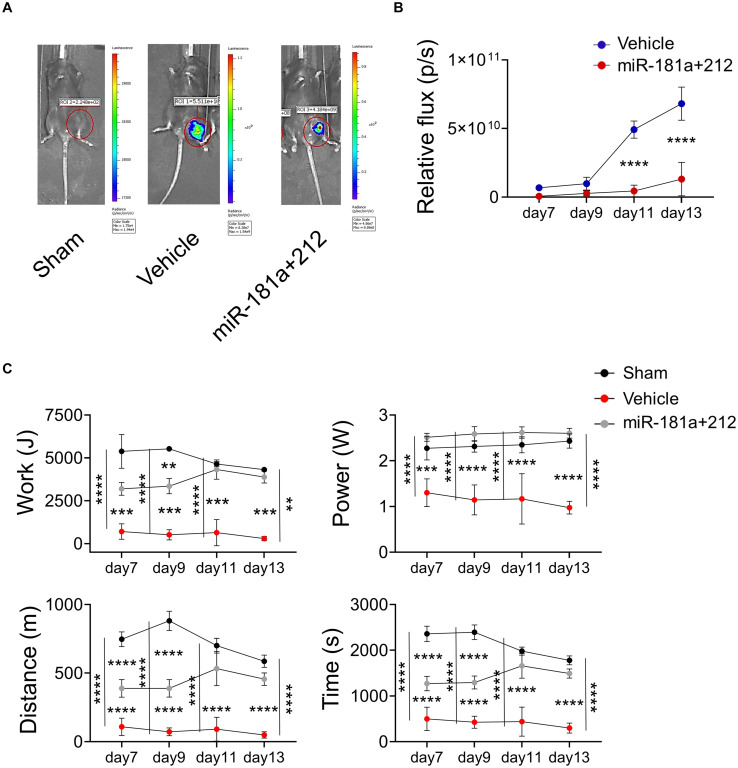
miR181a/miR212 effects on murine FN-RMS proliferative capacity *in vivo*. **(A)** Representative bioluminescent images of C57/Bl6 mice injected in the femoral artery with saline (control) or 1 × 10^5^ KMR46 Fluc+ cells (untreated group) or 1 × 10^5^ KMR46 Fluc+ cells pretreated for 3 days with miR-181a/212 (treated group). Images were taken 7-, 9-, 11-, and 13-days post-injection. **(B)** Quantification of the BLI signal (*n* = 3). **(C)** Functional test by treadmill exhaustion test at 7-, 9-, 11-, and 15-days post-injection. From left to right and top to bottom, graphs of work (J), power (W), distance (m), and time of run (min). (*n* = 3). **p < 0.01; ***p < 0.001; ****p < 0.0001.

Next, we assessed the effect of miR-181a/212 treatment in FN-RMS in terms of tumor progression *in vivo* and possible implication in impaired physical performance. Thus, we transduced murine FN-RMS cell line with lentiviral vectors carrying fLuc to monitor its activity *in vivo* (Supplementary Figure 3), and pretreated the cell line with the miRNAs before syngeneic transplants. Twelve C57/Bl6 mice were introduced to the treadmill belt in training sessions to make them familiar with the treadmill and tasks. Then the mice were injected in the femoral artery with saline (control) or 1 × 10^5^ KMR46 Fluc+ cells (untreated group) or 1 × 10^5^ KMR46 Fluc+ cells pretreated for 3 days with miR-181a/212 (treated group). Bioluminescent images revealed that untreated and treated groups showed already different amount of cells at day 7 from transplantation (Figure 4A) while no signal was found in mice injected in the femoral artery with saline (control). From day 11 the amount of cells increased dramatically in the untreated group where up to 6.8 × 10^10^ cells were detected at day 13 (Figure 4B). Functional assays were performed at day 7, 9, 11, and 13 after cell injections in trained mice. The mice injected with miRNA-pretreated cells showed an increment in functional outcome and they were able to run for significantly longer period and distance when compared with mice injected with untreated cells (Figure 4C). The better performance was even more pronounced at day 11 and 13 from injections in concomitance with the increased of tumor cell numbers in tumor-bearing mice injected with untreated cells (tumor masses were visible only in this group of mice). Other parameters of treadmill exhaustion test such as work and power showed similar statistically significant differences among the groups and in mice injected with miRNA-pretreated cells at day 11 and 13 those values were similar to sham-operated group (Figure 4C).

## Discussion

The majority of FN-RMSs exhibit embryonal tumor histology differently from FP-RMS characterized by the PAX3-FOXO1 fusion protein and alveolar histology (Tiffin et al., 2003; Linardic, 2008; Davicioni et al., 2009). Emerging literature identified miRNA dysregulation in FN-RMS cells compared to healthy muscle cells involved in the inhibition of adhesion independent growth and migration. For example, miR-22 and miR-378 family members are implicated in controlling proliferation and migration of FN-RMS cells through AKT/mTOR axis (Megiorni et al., 2014; Bersani et al., 2016). Thus, miRNAs have a crucial role in both suppressing the tumorigenicity of RMS and regulating paracrine pathway signaling responsible for tumor invasion and metastatic potential. Indeed, it is generally accepted that exosomal miRNA contents reflect the characteristic miRNA of the cells releasing the extracellular vesicles. However, several reports showed that in case of RMS, a modulation of exosomal miRNA exists compared with tumor cell content. In these circumstances, it could be possible that immune and inflammatory cells, which occur coincidentally at tumor lesions, could contribute to these exosomal miRNAs enrichment (Ghayad et al., 2016).

We and other groups showed that the potential of miR-based orchestration of cell fate is evident along the striated muscle lineages and miRNA mixtures have been described as part of the epigenetic signature retained after cell reprogramming in induced pluripotent stem cells. Thus, since FN-RMS cells are sharing some of gene expression profile with the immature mesodermal progenitors, we sought to test the PMC on FN-RMS cells to rewire the myogenic epigenetic memory similarly observed in treated pluripotent stem cell derivatives. Subsequently, a s-PMC reductionist approach reveals that the exposures to miR-181a and miR-212 only are sufficient to exert the FN-RMS tumor suppressive functions. miR-181a family is highly conserved and regulates many crucial biological processes including proliferation, mitochondrial function, apoptosis, and autophagy (Ouyang et al., 2012). In addition, some authors reported that they are dysregulated also in Alzheimer’s and Parkinson’s diseases (Indrieri et al., 2020). Albeit our s-PMC cocktail was tailored to murine FN-RMS, we confirmed that it is still able to upregulate Tnnt1 and Myl6, and downregulate Bmp6 as previously shown for PMC cocktail by Giacomazzi et al. (2017), while other direct target genes (Gdf15, Anxa3, Hand1, and Myo1d) are slightly affected. Although additional studies are required to pick up the most effective therapeutic miRNA cocktail, our data points on those targeted genes able to slow down RMS progression preserving muscle performance in mice injected with miR-181a and miR-212 pre-treated FN-RMS cells.

Similar to miR-181a, the abnormal expression of miR-212 may cause a series of neurodegenerative diseases, such as Alzheimer’s disease, epilepsy and schizophrenia. More recent studies show that miR-212 is involved in various forms of cancers and can act as a diagnostic or predictive marker of many tumors, including hepatocellular carcinoma ovarian cancer and gastric cancer (Chen et al., 2020). It is not surprising that as in non-small cell lung cancer, ovarian prostate also in FN-RMS act as tumor suppressors and play a substantial role in inhibiting tumorigenesis and FN-RMS progression. This last part is further strengthened by the fact that a remarkable reduction in BLI signal was observed in mice injected with FN-RMS Fluc+ cells pretreated for three days with miR-181a/212. In addition, the evaluation of muscle performance obtained by treadmill exhaustion test at up 13 days post-injection supported the beneficial effect of s-PMC pretreatments in injected mice. We can hypothesize that this beneficial effect would not last for long period since the tumor mass was starting to grow at day 13 even after the miRNA exposure. This is indeed the main limitation of our study that did not explore the effects of multiple miRNA treatment or the stable overexpression of the miRNAs in FN-RMS cells. Nevertheless, we can conclude that as observed in other form of tumors (Chou et al., 2013; Otoukesh et al., 2020) the perturbation of miRNA signature in FN-RMS cells hampered its proliferation and migration ability *in vitro* and *in vivo* with consequence in tumor progressions. Both histone modification and DNA hypermethylation can be responsible for the dysregulation of miR-181a/212 in different cancers and their modulation can be used as important biomarker for the diagnosis or prognosis of cancer since they can be easily detected in serum and plasma. The controversial findings regarding their participation in chemoresistance and radioresistance require further studies (Zhang et al., 2010; Taketo et al., 2018). In future experiments it would be necessary to investigate which of the predicted miR-181a and miR-212 gene targets are implicated in the beneficial effect observed in this study and eventually their involvement in the resistance to chemotherapy or radiotherapy will become an important focus in the field of cancer therapy. High-throughput studies are also crucial to identify the precise signature of FN-RMS at single cell level in order to identify dysregulated miRNAs in dormant tumor cells responsible for cancer relapse and drug resistance. It is widely accepted that patient-derived 3D tumoroids better approximate the *in vivo* physiology and microenvironment, thus providing remarkable tool for miRNA combination screening that allows the assessment of potential therapeutic agents and mechanistic studies.

## Data Availability Statement

The datasets presented in this study can be found in online repositories. Data has been deposited in GEO under accession code GSE175816.

## Ethics Statement

The animal study was reviewed and approved by the Ethical Committee KU Leuven for Animal Experimentation. Biomedische Wetenschappen, Animalium Gasthuisberg O&N 1 – Herestraat 49 – bus 501, B-3000 Leuven. ECD #P089/2018.

## Author Contributions

EP, NG, and MS designed the study and wrote the manuscript. EP, NG, ME, GS, C-CW, VM, and FR performed the experiments. EP and TK performed the bioinformatics analysis. EP and NG interpreted the data. JY and AU supervised the final manuscript. All authors have read and agreed to the published version of the manuscript.

## Conflict of Interest

The authors declare that the research was conducted in the absence of any commercial or financial relationships that could be construed as a potential conflict of interest.

## Publisher’s Note

All claims expressed in this article are solely those of the authors and do not necessarily represent those of their affiliated organizations, or those of the publisher, the editors and the reviewers. Any product that may be evaluated in this article, or claim that may be made by its manufacturer, is not guaranteed or endorsed by the publisher.

## Footnotes

1
http://www.stat.ubc.ca/~rollin/stats/ssize/index.html

## Supplementary Material

Supplementary Figure 1 s-PMC and s-AMC effect on Myog and Myod expression. Immunofluorescence of murine FN-RMS after s-PMC or s-AMC treatment compared to vehicle, showing reduced or increased Myogenin (red) and Myod (green) expression after AMC or PMC, respectively. Nuclei are counterstained in blue with HOECHST. Scale bar, 100 μm.

Supplementary Figure 2 *In silico* prediction of s-PMC pathways and selection of miR-181a/212. **(A)** DIANA-miRPath prediction of the GO pathways targeted by the single s-PMC miRNAs, and **(B)** by miR-181a/212 selection.

Supplementary Figure 3 Creation of KMR46-EF1a-GFP-fLuc cell line. *In vitro* validation of the insertion of the cassette by checking **(A)** GFP expression and **(B)** fLuc activity upon Renilla addition.

Supplementary Table 1 Complete list of s-PMC targets predicted by miRDB.

Supplementary Table 2 Complete list of s-PMC targets predicted by miRDB upregulated in FN-RMS.
