# Supplementary figures and images for: Upregulation of miR181a/miR212 Improves Myogenic Commitment in Murine Fusion-Negative Rhabdomyosarcoma
Source: Front Physiol. 2021 Aug 6;12:701354. doi: 10.3389/fphys.2021.701354 (PMC8378536; doi:10.3389/fphys.2021.701354)

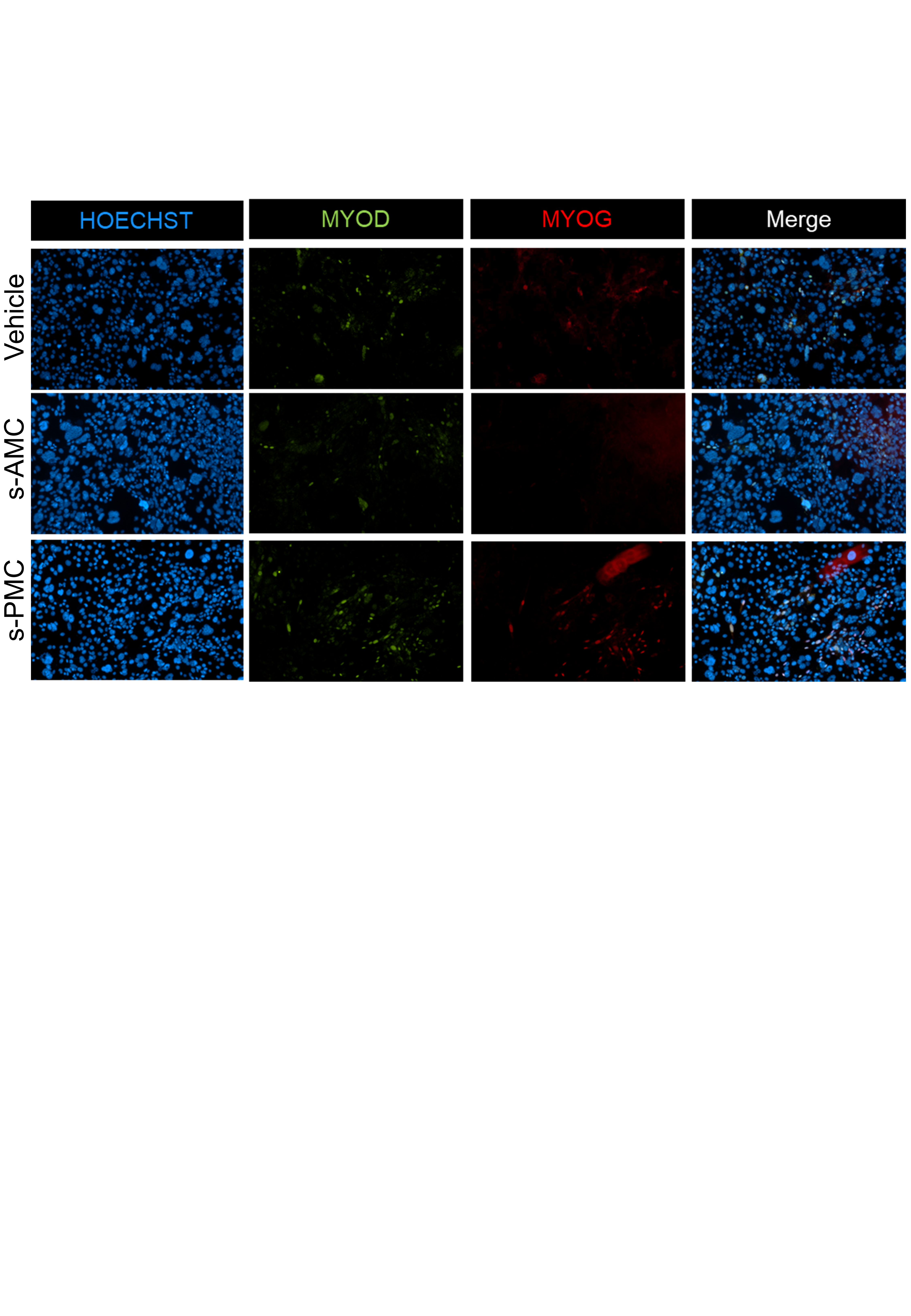

Supplement: Supplementary Figure 1 — s-PMC and s-AMC effect on Myog and Myod expression. Immunofluorescence of murine FN-RMS after s-PMC or s-AMC treatment compared to vehicle, showing reduced or increased Myogenin (red) and Myod (green) expression after AMC or PMC, respectively. Nuclei are counterstained in blue with HOECHST. Scale bar, 100 μm. [file Image_1.TIF]

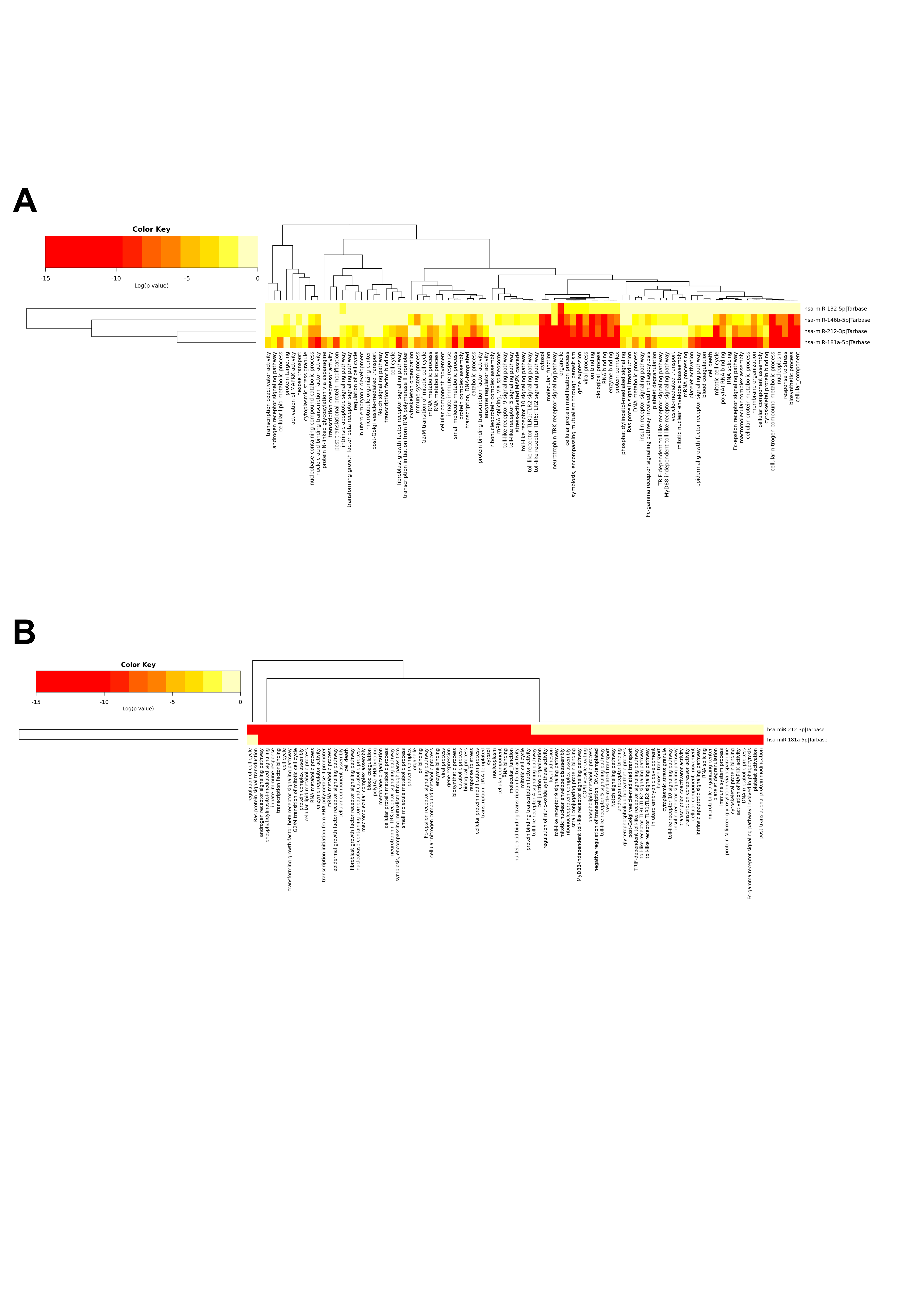

Supplement: Supplementary Figure 2 — In silico prediction of s-PMC pathways and selection of miR-181a/212. (A) DIANA-miRPath prediction of the GO pathways targeted by the single s-PMC miRNAs, and (B) by miR-181a/212 selection. [file Image_2.TIF]

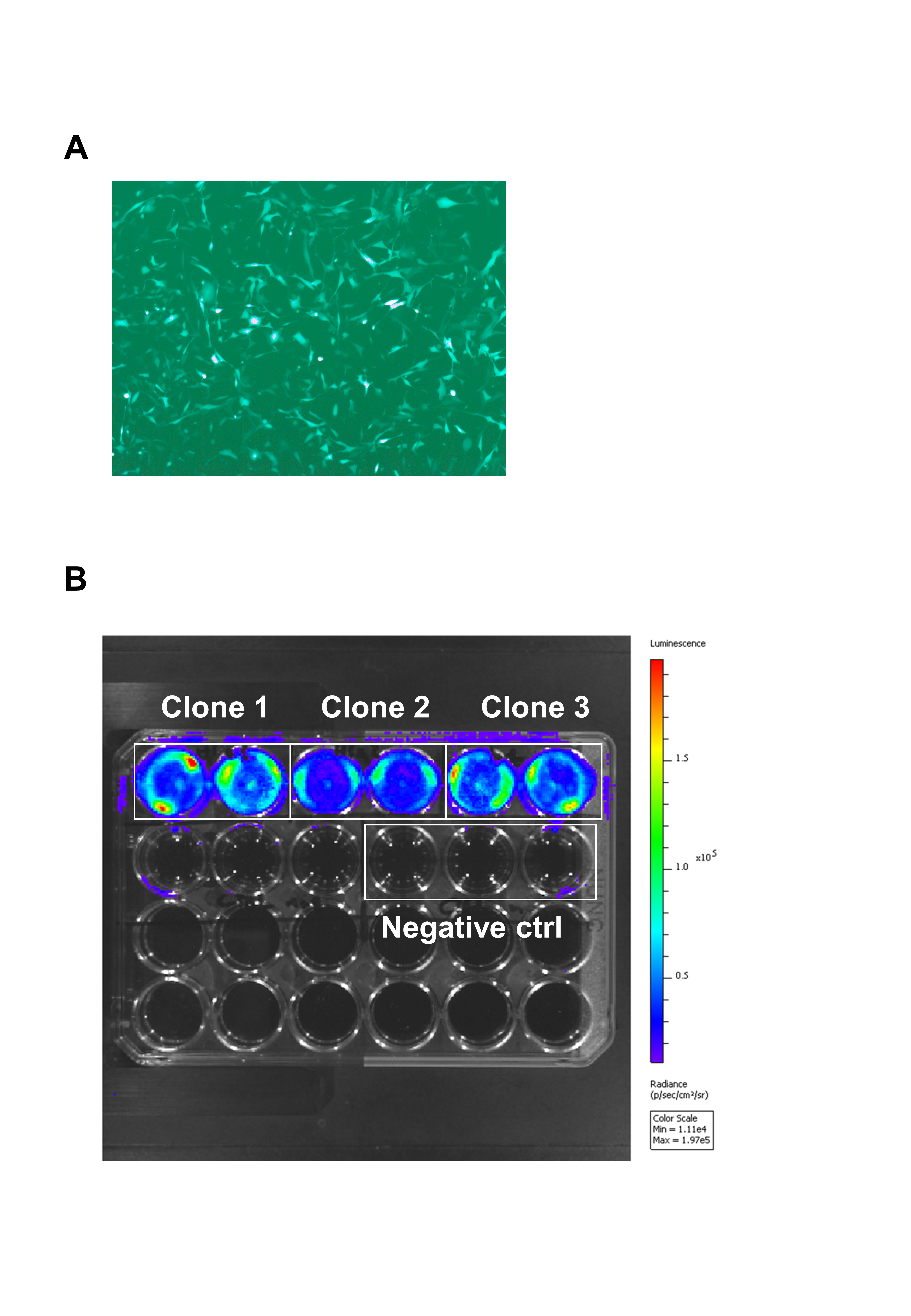

Supplement: Supplementary Figure 3 — Creation of KMR46-EF1a-GFP-fLuc cell line. In vitro validation of the insertion of the cassette by checking (A) GFP expression and (B) fLuc activity upon Renilla addition. [file Image_3.TIF]
